# Ventricular fibrillation treated by cryotherapy to the right ventricular outflow tract: a case report

**DOI:** 10.1186/s13256-016-1032-2

**Published:** 2016-09-15

**Authors:** Paramdeep S. Dhillon, Giulia Domenichini, Hanney Gonna, Anthony Li, Nadia Sunni, Michael Mahmoudi, Mark M. Gallagher

**Affiliations:** 1 Cardiology Clinical Academic Group, St. George’s University Hospitals NHS Foundation Trust, Blackshaw Road, London, SW17 0QT UK; 2 St Peter’s Hospital, Guildford Road, Chertsey, KT16 0PZ UK; 3 Department of Cardiology, St. Georges Hospital, Blackshaw Road, London, SW17 0QT UK

**Keywords:** Ventricular extrasystole, Right ventricular outflow tract, Cardiac arrest, Cryoablation, Case report

## Abstract

**Background:**

Arrhythmias originating from the right ventricular outflow tract are generally considered benign but cases of cardiac arrest have been described, usually associated with polymorphic ventricular tachycardia or extrasystoles with short coupling intervals.

**Case presentation:**

We report the case of a 54-year-old Caucasian woman with symptomatic right ventricular outflow tract arrhythmias without structural heart disease who suffered a ventricular fibrillation arrest without prior malignant clinical features. Cryoablation was performed and an implantable cardioverter defibrillator was implanted. She has since been free of arrhythmia for 7 years and has asked that the implantable cardioverter defibrillator not be replaced when the battery becomes depleted.

**Conclusions:**

Although usually benign, right ventricular outflow tract tachycardia can be life-threatening. Even the most malignant cases can be cured by ablation.

## Background

The right ventricular outflow tract (RVOT) is the commonest site of origin of frequent ventricular ectopic beats (VEB) and nonsustained ventricular tachycardia (NSVT) in patients with a structurally normal heart. These arrhythmias are generally benign. Cases of cardiac arrest have been described, but almost always in association with polymorphic ventricular tachycardia or extrasystoles with short coupling intervals.

We describe a case in which RVOT ectopy was complicated by ventricular fibrillation (VF) arrest despite the absence of any of the recognized risk factor for cardiac arrest and despite an apparently positive response to pharmacological therapy. At 7 years after cryoablation for the ventricular arrhythmia and implantation of an implanted cardioverter defibrillator (ICD), our patient has had no further significant ventricular arrhythmia. This case illustrates the limitation of current methods for predicting serious arrhythmias and for treating them pharmacologically.

## Case presentation

A 54-year-old Caucasian woman therapist with no past history of serious illness was referred for Holter monitoring in September 2008 after complaining of episodic palpitations over 4 years, resulting in disruption of daily activities. There was no history of syncope and no family history of arrhythmia or sudden death. At the time of presentation, she was taking no medication. Routine biochemistry and hematological analysis proved normal. A physical examination was normal.

A 24-hour Holter electrocardiogram (ECG) demonstrated frequent VEB, couplets and frequent runs of 3–5 beats of monomorphic NSVT. In total 2448 ectopic beats were present, all of the same morphology, with a relatively narrow ventricular ectopic QRS interval (VEQSI) of 160 ms. Transthoracic echocardiography demonstrated a structurally normal heart, confirmed by cardiac magnetic resonance scan. Our patient declined curative ablation. Because of a history of suspected asthma, beta-blocking drugs were relatively contraindicated. She was discharged on a slow-release preparation of verapamil 240 mg daily for control of symptoms. This reduced the frequency of ventricular ectopy and diminished the symptoms to a tolerable level.

**Fig. 1:**
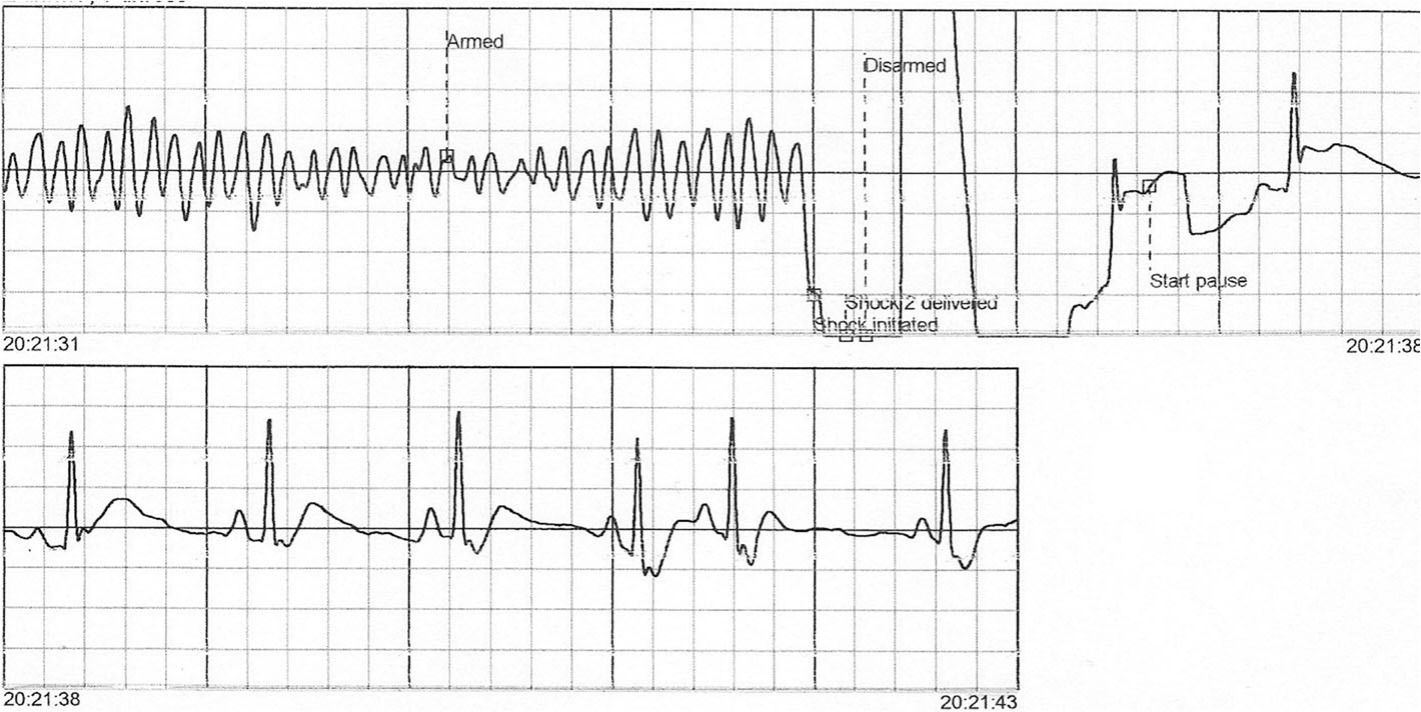
Electrographic traces showing the successful defibrillation of ventricular fibrillation

One month later, our patient was readmitted after suffering an out-of-hospital VF cardiac arrest from which she was successfully resuscitated (Fig. 1). Coronary angiography demonstrated normal coronary arteries. On recovery, our patient accepted our recommendation that she undergo ablation of the focus of ventricular ectopy but she refused implantable cardioverter defibrillator (ICD) therapy.

At electrophysiology study, a decapolar diagnostic electrophysiological catheter (Bard, Electrophysiology Division, Lowell, MA, USA) and a cryoablation catheter (Freezor Extra™, Medtronic, Minneapolis, MN, USA) were used. We found electrogram amplitude to be normal throughout the right ventricle. Frequent ventricular ectopy was present; activation mapping traced the site of origin to the anterior aspect of the RVOT approximately 1 cm below the pulmonary valve.

**Fig. 2:**
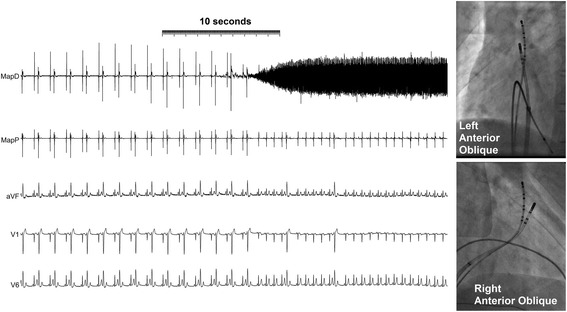
Elimination of ventricular ectopy by cryoablation. Ectopic beats occurring consistently every second beat are eliminated within 10 seconds of the cooling of the ablation catheter to –80 °C as demonstrated by the cooling artefact on the ablation D bipolar electrogram. Radiological images of the cryoablation catheter at the site of successful ablation are shown on the *right*

Cryotherapy to –80 °C for 3 minutes was delivered at this site with complete suppression of ectopy during delivery but recurrence on rewarming. A second delivery at an adjacent site resulted in persistent elimination of ectopy (Fig. 2). No further ventricular ectopy occurred during 40 minutes of observation in the electrophysiology laboratory or during the 4 days of that hospital admission.

Holter recordings 1 day and 1 week after ablation recorded only three ectopic beats on each occasion, none of which appeared to be of the clinical morphology. After reflection, our patient decided to accept ICD therapy. A single-lead ICD was implanted 10 days post-ablation. At 7.5 years post-ablation, interrogation of her ICD shows no record of any sustained ventricular arrhythmia. Our patient has requested that the device not be replaced when the battery becomes depleted.

## Discussion

We report a case of cardiac arrest in a patient with frequent VEB and NSVT originating in the RVOT of a structurally normal heart. This was an unusual presentation in that no high-risk feature was present such as preceding symptoms of syncope [1], short coupling interval of the VEB [2], or rapid ventricular tachycardia (VT) [2], which have been previously reported, nor did the extrasystoles fall within the preceding T wave [3]. The coupling interval of our patient’s extrasystole was 448 ± 22 ms in comparison with a mean of 340 ± 30 ms reported for patients developing polymorphic VT in a series reported by Viskin *et al.* [2]; the cycle length during episodes of nonsustained VT was approximately 280 ms in comparison with a mean of 245 ± 28 ms reported for patients with polymorphic VT and VF by Noda *et al.* [1]*.*

The fact that we suppressed ectopy and sustained ventricular arrhythmia using ablation at a single site suggests that her heart is otherwise normal. Similar results have been described in the series of Noda *et al.*, where 16 patients with RVOT extrasystole-related polymorphic VT or VF underwent successful radiofrequency ablation targeting the initiating extrasystoles which eliminated syncope and cardiac arrest during follow-up of 54 ± 39 months [1].

In this case we used cryoablation to avoid the pain associated with radiofrequency delivery and hence the possibility of suppression of ectopy by the autonomic activation arising from that pain or from the medications needed to control it. The complete and persistent elimination of previously very frequent VEB by cryoablation at a single site in the absence of sedation provided compelling evidence that the electrophysiological abnormality was limited to that site.

We recommended ICD therapy in this case in accordance with most guidelines following the AVID study [4]. This trial showed that ICD therapy is clearly superior to medical therapy alone in patients who have survived a cardiac arrest. We have attributed this patient’s cardiac arrest to the underlying arrhythmia because of the lack of any evidence of any other cardiac condition after the extensive investigations listed and the short VEQSI recorded [5, 6]. We consider it extremely unlikely to have been due to pro-arrhythmia related to the use of verapamil based on the lack of clinical evidence of such a pro-arrhythmic effect, the laboratory evidence against the existence of such an effect [7], and the lack of any pro-arrhythmic effect on the Holter monitoring of this patient between the time of initiation of verapamil therapy and the occurrence of the cardiac arrest.

Although the superiority of ICD therapy over medical therapy alone is clearly demonstrated, the relative efficacy of ablation compared to ICD therapy has not been quantified accurately. In the series of Noda *et al.*, ICD therapy was not used and no patient died, but the total follow-up time in that cohort was less than 75 patient-years. Other studies have demonstrated the ability of ablation to prevent recurrence of VF in patients with a structurally normal heart [8], but these studies also have been of small patient cohorts followed only for a few years. Until the effectiveness of ablation for the prevention of VF has been confirmed in a large cohort of such patients followed for a substantial duration, we will continue to recommend ICD therapy for most survivors of VF arrest even if ablation is also performed.

## Conclusions

Arrhythmias originating in the RVOT, although benign in most patients, may result in cardiac arrest. The absence of a history of syncope, and the presence of long-coupled extrasystoles and a long cycle of nonsustained VT, does not exclude the possibility of life-threatening ventricular arrhythmias. Even malignant forms of RVOT arrhythmia are amenable to successful catheter ablation.

## Abbreviations

ICD: Implanted cardioverter defibrillator
NSVT: Nonsustained ventricular tachycardia
RVOT: Right ventricular outflow tract
VEB: Ventricular ectopic beat
VEQSI: Ventricular ectopic QRS interval
VF: Ventricular fibrillation
VT: Ventricular tachycardia
